# The Influence of Heterochronic Non-Myeloablative Bone Marrow Transplantation on the Immune System, Frailty, General Health, and Longevity of Aged Murine Recipients

**DOI:** 10.3390/biom12040595

**Published:** 2022-04-18

**Authors:** Katerina Jazbec, Mojca Jež, Urban Švajger, Boštjan Smrekar, Simona Miceska, Uroš Rajčevič, Mojca Justin, Janja Završnik, Tadej Malovrh, Tanja Švara, Mitja Gombač, Živa Ramšak, Primož Rožman

**Affiliations:** 1 Diagnostic Services, Blood Transfusion Centre of Slovenia, 1000 Ljubljana, Slovenia; mojca.jez@gmail.com (M.J.); urban.svajger@ztm.si (U.Š.); bostjan.smrekar@ztm.si (B.S.); miceska.simona@gmail.com (S.M.); urajcevic@yahoo.com (U.R.); mojca.justin90@gmail.com (M.J.); primoz.rozman@ztm.si (P.R.); 2 Chair of Clinical Chemistry, Faculty of Pharmacy, University of Ljubljana, Aškerčeva 7, 1000 Ljubljana, Slovenia; 3 Department of Biochemistry and Molecular and Structural Biology, Jožef Stefan Institute, 1000 Ljubljana, Slovenia; janja.zavrsnik@drustvo-vzd.si; 4 Institute of Microbiology and Parasitology, Veterinary Faculty, University of Ljubljana, 1000 Ljubljana, Slovenia; tadej.malovrh@vf.uni-lj.si; 5 Institute of Pathology, Wild Animals, Fish and Bees, Veterinary Faculty, University of Ljubljana, 1000 Ljubljana, Slovenia; tanja.svara@vf.uni-lj.si (T.Š.); mitja.gombac@vf.uni-lj.si (M.G.); 6 National Institute of Biology, 1000 Ljubljana, Slovenia; ziva.ramsak@nib.si

**Keywords:** bone marrow transplantation, BMT, immune system, aging, mice, hematopoietic stem cells, cell isolation, frailty index, longevity

## Abstract

The stem cell theory of aging postulates that stem cells become inefficient at maintaining the original functions of the tissues. We, therefore, hypothesized that transplanting young bone marrow (BM) to old recipients would lead to rejuvenating effects on immunity, followed by improved general health, decreased frailty, and possibly life span extension. We developed a murine model of non-myeloablative heterochronic BM transplantation in which old female BALB/c mice at 14, 16, and 18(19) months of age received altogether 125.1 ± 15.6 million nucleated BM cells from young male donors aged 7–13 weeks. At 21 months, donor chimerism was determined, and the immune system’s innate and adaptive arms were analyzed. Mice were then observed for general health and frailty until spontaneous death, when their lifespan, post-mortem examinations, and histopathological changes were recorded. The results showed that the old mice developed on average 18.7 ± 9.6% donor chimerism in the BM and showed certain improvements in their innate and adaptive arms of the immune system, such as favorable counts of neutrophils in the spleen and BM, central memory Th cells, effector/effector memory Th and Tc cells in the spleen, and B1a and B1b cells in the peritoneal cavity. Borderline enhanced lymphocyte proliferation capacity was also seen. The frailty parameters, pathomorphological results, and life spans did not differ significantly in the transplanted vs. control group of mice. In conclusion, although several favorable effects are obtained in our heterochronic non-myeloablative transplantation model, additional optimization is needed for better rejuvenation effects.

## 1. Introduction

Immunosenescence, aging of the immune system, leads to greater susceptibility to infections, greater likelihood of autoimmune and inflammatory diseases, and cancer. Europeans in the over-65 age group are most likely to die from heart disease, respiratory disease, cerebrovascular disease, and cancer (Eurostat, 2019 [1]). Several authors have already described the detrimental role of aging on immunity in the early 70ies. A series of elegant animal and in vitro experiments documented the decline of humoral and cell-mediated immunity in aged animals, which appear as early as when an individual reaches sexual maturity. They documented the decline in T cell functions affecting the entire immune system, changes in antibody formation and B cell functions, an increase of autoantibodies in older experimental animals and humans, and enhancement of malignant development, which were all mediated by loss or shift in the proportion of immune cell subpopulations, qualitative cellular changes, involution of the thymus, or diminished capacity of stem cells in aged organisms [2,3,4,5,6,7].

Therapeutic interventions aimed at maintaining the immune system could delay or even prevent age-related diseases associated with weakened immunity, reduce overall frailty, prolong life expectancy, and enable healthy aging [8,9].

The stem cell theory of aging assumes that the inability of different types of stem cells to successfully regenerate tissues with a sufficient number of properly functional differentiated cells is responsible for the tissue and organ aging [10]. Aging significantly reduces the therapeutic potential of stem cells, which is evident both in stem cells isolated from the elderly and in stem cells that had been cultured for many passages in vitro [11].

All immune and blood system cells are formed from hematopoietic stem cells (HSCs) in the bone marrow (BM). Like all somatic stem cells, HSCs are subjected to the effects of aging [11,12]. The aging process of HSCs could be temporarily stopped by isolating and cryopreserving them at a young age and then returning them to the body when old age, the process called heterochronic autologous HSC transplantation (haHSCT). In this way, young cryopreserved HSCs could, in theory, renew and improve the functioning of the immune and hematopoietic system in old age in an autologous manner and prolong healthspan, i.e., the period of life spent in good health, free from chronic diseases and disabilities of aging [13,14].

The purpose of this study was to develop an experimental murine model of haHSCT and investigate whether young HSCs could favorably modulate the functioning of the old immune system, decrease frailty, and perhaps extend the lifespan. We conducted a study on mice, which were chosen due to their short lifespan and their history of being an appropriate model for studying the effects of HSC or bone marrow transplantation (BMT). Since a completely autologous setting is practically unattainable in mice due to the scarcity of biological materials of an individual mouse, we used a syngeneic heterosexual donor/recipient combination to closely mimic the autologous tissue compatibility setting [15,16,17]. Old female BALB/c mice as recipients, and young inbred BALB/c males as donors were also chosen for enabling precise determination of the post-transplantation mixed chimerism, based on the presence of the Y chromosome, as already set in our previous studies [18,19]. Similarly, since the isolation of large numbers of pure HSCs from young mice needed for consequent long-term cryopreservation and transplantation is practically impossible, HSCT was substituted by transplantation of the whole BM of young donors.

## 2. Materials and Methods

### 2.1. Animals

BALB/c AnNCrl (H-2d) mice (19–25 g; ex breeders) were maintained in the animal facility at the Blood Transfusion Centre of Slovenia (Ljubljana, Slovenia) under specific pathogen-free conditions. Mice were housed on a 12-h light/dark cycle in standard group cages (5/cage), with ad libitum access to food (Rehofix MK 2000, Rettenmaier & Söhne, Rosenberg, Germany) and water, and with red houses and tunnels for enrichment. Donors of the BM cells were males aged from 7 to 13 weeks; recipients were females aged 14 months at the start of the experiment. Mice were ear punched for identification. The study was approved by the Ethical Commission for the Experiments on Animals (No. U34401-11/2014/6 from 11 April 2014, U34401-11/2014/13 from 20 March 2017, U34401-9/2015/5 from 14 April 2015, U34401-27/2015/7 from 10 December 2015 and U34401-27/2016/5 from 16 November 2016) from the Administration of the Republic of Slovenia for Food Safety, Veterinary and Plant Protection.

### 2.2. The Experimental Scheme

**Figure 1:**
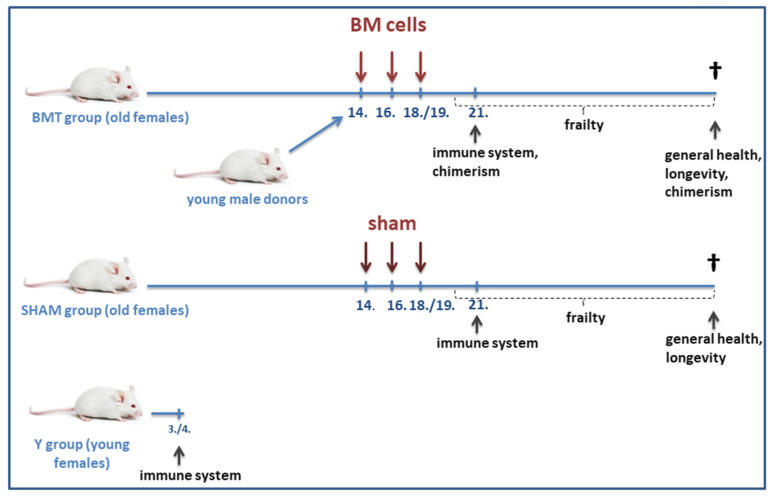
The experimental scheme. Numbers indicate the age of mice in months, “BM cells” indicate the time of BM transplantations, and “sham” the time of DPBS injections. Legend: BMT group—received the BMT, SHAM group—control group, and Y group—control group of young female mice.

At the age of 14, 16, and 18(19) months, the experimental group of old mice (BMT group; *n* = 60) received BM transplantations from young donor males, while the old control mice received sham transplantations (SHAM group; *n* = 41). At 21 months, a part of the animals in each group (*n* = 15 for immunophenotyping and in vitro tests, *n* = 10 for in vivo test) was sacrificed for immune system testing. Another control group of young mice (*n* = 15 for immunophenotyping and in vitro tests, *n* = 10 for in vivo test) served for the observation and comparison of aging effects (Figure 1).

### 2.3. Heterochronic Non-Conditioned Bone Marrow Transplantation

The non-conditioned female recipient mice were injected 10–20 × 10^6^ nucleated BM cells in a final volume of 200 μL DPBS (Gibco, Bleiswijk, The Netherlands) into the tail vein. BM cells from young male donors were injected on three occasions per week at 14, 16, and 18/19 months of age, constituting on average eight (8) injections. The cell suspension was freshly prepared for each injection, and the final cell suspension was filtered through a 30-μm nylon cell strainer (Miltenyi Biotec, Bergisch Gladbach, Germany). The total amount of transplanted BM cells was on average 125.1 ± 15.6 million cells per animal.

### 2.4. Bone Marrow Cell Isolation

As previously reported by [18,20], the mice were sacrificed using CO_2_ asphyxiation and cervical dislocation, and the main bones (femurs, tibias, humeri, ilia, and spine) were dissected and thoroughly cleaned. Bones were crushed in cold RPMI+ medium (RPMI-1640 medium (Gibco, Bleiswijk, The Netherlands), supplemented with 25 mM HEPES (Gibco, Bleiswijk, The Netherlands), 300 mg/L L-glutamine (PAA, Cambridge, UK), 1 mM EDTA (Gibco, Bleiswijk, The Netherlands), and 1× PenStrep (Gibco, Bleiswijk, The Netherlands), using a sterile mortar and pestle. The cell suspension was filtered through a 40 µm nylon cell strainer (Falcon, Corning, NY, USA) twice and centrifuged for 5 min at 490× *g* at 4 °C. Red blood cell lysis was performed using 1× PharmLyse Buffer (BD Biosciences, San Jose, CA, USA) and washed twice with DPBS (Gibco, Bleiswijk, The Netherlands).

### 2.5. Cell Isolation from the Peritoneal Cavity

Cells were isolated from the peritoneal cavity using an adapted protocol from Ray and Dittel [21]. Briefly, we carefully cut through the outer skin of the abdominal region and gently pulled it back to expose the inner muscle lining the peritoneal cavity. We injected 5 mL of cold RPMI+ medium into the peritoneal cavity using a 25 g needle. After injection, we gently massaged the peritoneum and collected the fluid with the needle. Then we cut and collected additional fluid using the Pasteur pipette. The collected fluid was centrifuged at 490× *g* for 5 min, and the pellet was resuspended in DPBS (Gibco, Bleiswijk, The Netherlands). The cell suspension was discarded if it had visible blood contamination.

### 2.6. Cell Isolation from Spleen

Splenocytes were isolated in a cold RPMI+ medium. The spleen was put in a 40 µm nylon cell strainer (Falcon, Corning, NY, USA) in a Petri dish with a few ml of medium. Spleen was gently crushed using a syringe plunger until only connective tissue was left. The cell suspension was centrifuged at 450× *g* for 5 min at 4 °C, and red blood cell lysis was performed using 1× PharmLyse Buffer (BD Biosciences, San Jose, CA, USA) and washed twice with DPBS (Gibco, Bleiswijk, The Netherlands).

### 2.7. Phenotypic Characterization with Flow Cytometry and Fluorescence-Activated Cell Sorting

For phenotypic cell characterization with flow cytometry and fluorescence-activated cell sorting, cells were stained with antibodies Ly6G-PE, Ly6C-APC, CD11b-PE-Vio770, CD150(SLAM)-APC, CD48-PE, CD41-FITC, Lin-PerCP-Cy5.5, c-Kit (CD117)-PE-Vio770, B220(CD45R)-APC-Vio770, CD19-PE-Vio770 or APC-Vio770, CD43-PE, surf. IgM-FITC, surf. IgD-APC, CD21/35-FITC, CD23-APC, AA4.1(CD93)-PE-Vio770, CD5-PE, CD3-APC-Cy7, CD4-PerCP-Cy5.5 or FITC, CD8a-FITC, CD44-PE, CD62L-APC, FoxP3-PE, CD25-APC, CD19-PE-Vio770, together with e-Fluor or 7AAD viability dye (Miltenyi Biotec, Bergisch Gladbach, Germany, BD Biosciences, San Jose, CA, USA, and eBioscience, San Diego, CA, USA), and analyzed with a flow cytometer (FACSAria I; BD Biosciences, San Jose, CA, USA). The appropriate flow cytometry controls were applied throughout and included unstained controls, fluorescence minus one (FMO) controls, and the addition of Fc block (Miltenyi Biotec, Bergisch Gladbach, Germany) to prevent non-specific antibody binding. Instrument compensation and cell subset gates were set based on unstained and FMO controls, all based on viable forward-vs side-scatter gates. Doublets were excluded from the analysis. For gating strategies see Supplementary Figures S1–S6.

### 2.8. DNA Isolation

As previously reported by [18], the recipient’s BM cells from the tibias, femurs, and spine were isolated. The cell suspension was collected and DNA isolated using EZ1 DNA Blood 350 μL kit on BioRobot EZ1 (Qiagen, Hilden, Germany) and DNeasy Blood and Tissue Kit (Qiagen, Hilden, Germany) following the manufacturer’s instructions. The DNA concentration was measured with NanoDrop (Thermo Fisher Scientific, Waltham, MA, USA).

### 2.9. Chimerism Determination with Quantitative Real-Time PCR (qPCR)

For the determination of chimerism in BM, spleen, lungs, and sorted cells, we performed a customized qPCR protocol by An and Kang [22] as reported previously by [18]. Briefly, primers for the Y chromosome-specific Zfy1 gene were used to detect donor DNA, and primers for gene Bcl2 were used for the normalization. The reaction volume (10 µL) contained 400 nM of each primer (Applied Biosystems, Waltham, MA, USA), 10 ng of the genomic DNA, and 2× Power SYBR Green PCR Master Mix (Applied Biosystems, Waltham, MA, USA). Standards were set in triplicates and samples in duplicates. The qPCR protocol was performed using ViiaTM 7 (Life Technologies, Carlsbad, CA, USA). The standard curve was established from the known male/female standard mixtures.

### 2.10. Donor Chimerism in the Hematopoietic Colony-Forming Units

As previously reported by [18], to determine the percentage of hematopoietic colony-forming units (CFU) of donor origin, BM cells were seeded in a semi-solid mouse MethoCult medium (GF 03434, Stem Cell Technologies, Vancouver, BC, Canada) following the manufacturer’s instructions. Briefly, cells were plated at a density of 50,000 cells per 35-mm Petri dish. After 10–12 days, single colonies were randomly picked with 10 μL pipettes and resuspended in 200 μL of DPBS. After the colonies were vortexed and centrifuged for 10 min at 500× *g*, dry pellets were stored at −80 °C until use. To obtain DNA for PCR analysis, tubes containing single colonies were freeze-thawed 5 times in liquid nitrogen and warm water. For each mouse, we analyzed 28–34 colonies. Each 10 μL PCR contained 400 nmol/L of each primer (Applied Biosystems), PowerUp SYBR Green PCR Master Mix (Applied Biosystems, Waltham, MA, USA), and 2.5 μL of 3× diluted supernatants containing DNA. The PCR was performed using Viia™ 7 (Life Technologies, Carlsbad, CA, USA), and amplicons for Y chromosome-specific Zfy1 gene (80 bp) and Bcl2 (97 bp) were visualized using 2% agarose gel.

### 2.11. Endocytosis Studies

Endocytosis was evaluated with flow cytometry after incubating the cells with FITC-dextran (avg mol wt 40,000, Sigma Aldrich, St. Louis, MI, USA), either at 37 °C or at 4 °C for 1 h. The cells were then washed with cold DPBS, centrifuged at 490× *g* for 5 min, and resuspended in DPBS (Gibco, Bleiswijk, The Netherlands). They were stained with anti-CD11b-PE-Cy7, anti-Ly6G-PE, anti-Ly6C-APC, and 7-AAD viability dye (Miltenyi Biotec, Bergisch Gladbach, Germany) and analyzed with a flow cytometer (FACSAria I; BD Biosciences, San Jose, CA, USA).

### 2.12. Cytokine Release Assay

Cells isolated from the spleen and peritoneal cavity were incubated with the addition of LPS (20 ng/mL, lipopolysaccharide, Sigma-Aldrich) at 37 °C. After 48 h, duplicate supernatant aliquots were merged and thawed once. Cytokines, IL-6, TNF, and MPC-1, were measured with Cytometric Bead Array (CBA) Mouse Inflammation Kit (BD Biosciences, San Jose, CA, USA) according to the manufacturers’ instructions, and results interpolated from the standard reference curve provided with the kit.

### 2.13. Splenocyte Proliferative Response

The assays were carried out in 96-well plates, with a total volume of 280 µL per well. 2 × 10^5^ splenocytes were plated in quintuplicates. Cells were stimulated with PHA (2 µg/mL) or PHA plus PMA (50 ng/mL), while control cells were unstimulated. After 4 days of incubation at 37 °C, the wells were pulsed with 1 µCi/well 3H-thymidine (Perkin Elmer, Waltham, MA, USA), and proliferation was measured by its incorporation after 18 h by liquid scintillation counting.

### 2.14. In Vivo Immunization

38–42 days before the immune system testing, 10 mice per group were immunized with keyhole limpet hemocyanin (KLH). On day 1, the vaccine with 25 µL KLH (1 mg/mL; Imject Maleimide Activated mcKLH, Thermo Scientific, Waltham, MA, USA), 75 µL PBS, and 100 µL CFA (Complete Freund’s adjuvant, Sigma-Aldrich Company Ltd., St. Louis, MI, USA) was injected subcutaneously. On days 14 and 28 vaccines with 25 µL KLH (1 mg/mL; Imject Maleimide Activated mcKLH, Thermo Scientific, Waltham, MA, USA), 75 µL PBS, and 100 µL IFA (Incomplete Freund’s adjuvant, Sigma-Aldrich Company Ltd., St. Louis, MI, USA) was injected intraperitoneally. 10–14 days after the last immunization, blood was taken from the tail vein. Blood was centrifuged on 1460× *g* for 10 min, and serums were frozen at −20 °C until antibody production analysis.

### 2.15. Antibody Detection Analysis

The presence of antibodies in sera was checked by their capacity to bind KLH in ELISA. Microtiter plates (Nunc, Roskilde, Denmark) were coated with 50 µL of KLH in an ELISA coating buffer (pH 9.6), incubated overnight at 4 °C, washed, and then blocked with 1% bovine serum albumin (Sigma-Aldrich Company Ltd., Dorset, UK). Fifty microliters of sera diluted to 1:10,000, 1:100,000, and 1:1,000,000 were added to the wells and incubated for 90 min at 37 °C. Plates were washed, and 50 µL of horseradish peroxidase-conjugated anti-mouse immunoglobulin (Jackson ImmunoResearch, West Grove, PA, USA), diluted 1:5000, was added and incubated for 90 min at 37 °C. After a final wash, the 2,2′-azino-b (3-ethylbenzthiazoline-6-sulfonic acid)(ABTS; Sigma-Aldrich Company Ltd., Dorset, UK) substrate was added. Absorbance was measured at 450 nm on a reference filter 620 nm after 15 min of incubation at 37°. Levels of antibodies were evaluated by the end-point dilution approach (the highest dilution of the sera was still three times higher than the background).

### 2.16. Frailty Evaluation

The long-term effects of the heterochronic BMT were also evaluated with a frailty index (FI) developed by Whitehead et al. [23]. FI assesses health parameters in a simplified and non-invasive way. It is based on 31 parameters; we used 30 parameters, as we used only white mice (we omitted the criterion of coat color). A scale (with an accuracy of 0.01 g), clicker, tweezers, and a thermometer (Braun PRO 4000 ThermoScan, Braun, Kronberg im Taunus, Germany) were used. Briefly, we evaluated the 30 parameters with the scoring system (0, 0.5, or 1) described by Whitehead et al. [23]. The mean scores (±SD) were calculated for each mouse.

### 2.17. Pathomorphological Investigation

Full necropsy including the evaluation of observed pathoanatomical changes was performed immediately after the death of a single mouse. Besides, organs and tissue samples were collected for histopathological, bacteriological, parasitological, and viral examination. For histopathological examination, samples of urethra, liver, gallbladder, skin, sternum, abdominal aorta, ovary, and fallopian tubes, kidneys, adrenal glands, spleen, thymus, uterine body and cervix, trachea and esophagus, lungs, heart, femur, brain, a segment of the thoracic and lumbar spine, peritoneum, abdominal fat, stomach, small (duodenum, jejunum, and ileum) and large intestine (caecum, ascending and descending colon and rectum) and mesenteric lymph nodes were collected. All detected lesions including neoplasms were additionally sampled. Samples for pathohistological examination were immediately fixed in 10% buffered formalin for 24 h at room temperature and routinely embedded in paraffin. Four-µm thick tissue sections were deparaffinized, stained with hematoxylin and eosin (HE), and examined with a light microscope. The femur, tibia, and part of the thoracic and lumbar spine were placed in the DPBS (Gibco, Bleiswijk, The Netherlands), and the BM was isolated the same day to determine chimerism.

### 2.18. Longevity

Mice were sacrificed if they reached humane endpoints, which were adjusted to traits of aged animals. In mice, we observed their general appearance (appearance of fur, eyes, nose, mouth, and head), respiration, the formation of outgrowths, and movement/resting. We monitored their body weight and temperature, including respiratory problems, the most critical signs of imminent death in old mice [24].

### 2.19. Statistical Analysis

Quantitative data from our study are summarized by the mean and standard deviation. We performed a two-way analysis of variance (ANOVA) with the main factors group and immunization and their interaction. Corrections for multiple testing were performed using the Bonferroni method. The comparison between BMT/SHAM and SHAM/Y was performed by contrast analysis. For the cytokine release assay, non-parametric comparisons (the Wilcoxon rank-sum test) were performed; due to multiple comparisons, the adjusted *p*-value was calculated according to Holm’s method. We performed a nonparametric Kruskal-Wallis test for the ELISA. FDR Benjamini and Hochberg was performed for the comparison between the groups. Correlations were calculated using the Pearson correlation analysis. We performed a normal linear mixed model analysis for comparing the frailty index between BMT and SHAM groups.

Heatmaps were drawn using Pearson coefficients as a distance measure between samples, using the pheatmap package in R [25,26] using the source data in Table S1. For the frailty index, we performed an analysis of a normal linear mixed model and the chi-squared test for the analysis of pathophysiologic changes. To evaluate the effects of several variables on overall survival, Kaplan-Meier survival analysis (log-rank statistics) was performed. The hazard ratio was calculated using Cox regression analysis.

## 3. Results

### 3.1. Chimerism and HSC Number

The BALB/c mice (*n* = 60) received on average 125.1 × 10^6^ ± 15.6 × 10^6^ (± SD) BM cells, which led to 18.7 ± 9.6% chimerism in their BM. To confirm the proliferation of donor hematopoietic stem and progenitor cells (HSPCs) in the recipients’ BM, fluorescence-activated cell sorting of neutrophils as representatives of the myeloid lineage, and B and T cells as representatives of lymphoid lineage were performed. Sorted populations were analyzed for chimerism (*n* = 12). In neutrophils, the chimerism was 19.4 ± 8.7%, in B cells it was 17.9 ± 7.7%, and in T cells, it was 3.3 ± 1.6%. In other words, chimerism in the neutrophil and B cell population was similar to the chimerism in the general BM cell population, while in the T cell population, it was lower and very variable (from 0% to 9.4%).

To confirm the engraftment, viability, and differentiation potential of the engrafted HSPCs, a hematopoietic CFU assay was performed, where cells proliferated into burst-forming unit-erythroid (BFU-E), granulocyte-macrophage colony-forming unit (CFU-GM), and granulocyte, erythroid, macrophage, megakaryocyte colony-forming units (CFU-GEMM) [18]. Engraftment results at the age of 21 months show that 44.0 ± 12.5% (*n* = 5) of multipotent and lineage-committed hematopoietic progenitor cells of the CFU colonies were of male donor origin.

Besides the engraftment, we were interested in the possible expansion of the HSC number after the BMT. For this reason, we have also analyzed the HSC frequency in the BM, which are defined as Lin^−^c-Kit^+^, CD150^+^, CD48^−^, CD41^−^ cells. In 21–22 months old BMT mice (*n* = 14), we counted 8.64 ± 4.25 HSCs per 100,000 BM nucleated cells, whereas in SHAM mice (*n* = 14) we counted 7.57 ± 5.05 HSCs per 100,000 BM nucleated cells. There was no statistically significant difference in HSC numbers between the groups.

### 3.2. Analysis of the Immune System

Phenotypic flow cytometric analyses of the immune system were made with 7 different analysis panels, three (3) in vitro functional tests (endocytosis evaluation, cytokine release assay, and splenocyte proliferation response), and one (1) in vivo functional test (immunization with KLH). The effects of heterochronic BM transplantation were evident in twelve (12) analyzed parameters: ten (10) parameters showed changes indicating a positive impact (“rejuvenating effect”), and two (2) parameters showed the opposite (“adverse effect”) (Table 1, Table 2, Table 3, Table 4, Table 5, Table 6, Table 7 and Table 8).

#### Heatmap of the Immune Parameters

**Figure 2:**
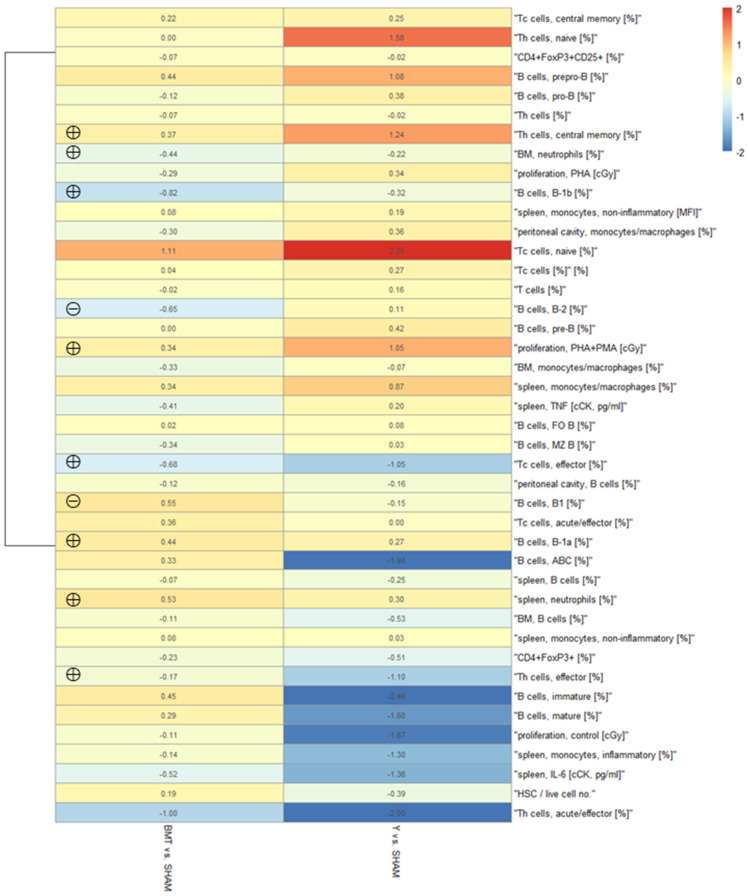
Heatmap of the analyzed parameters. The types of analysis are shown along the right axis, group comparison along the bottom axis. Red results indicate that the result is higher than in the SHAM group, and blue results indicate that the result is lower than in the SHAM group. Numbers in the boxes indicate the factor: 1 means the result is two times higher, and −1 indicates that the result is two times lower. Legend: BMT—transplanted aged mice, SHAM—aged control mice, Y—young control mice, ⊕ rejuvenating effect, ⊝ adverse effect.

A heatmap of normalized measured values of the BMT group compared to the SHAM control was drawn from the collected data of cell phenotype analyses and functional tests. Heatmap shows logarithmic (basis = 2) values of normalized measurements compared to the control (i.e., BMT vs. SHAM and SHAM vs. Y). The types of measurements are arranged according to the similarity of the Pearson coefficients, and the comparisons are made according to the Euclidean distance between them. Measured values with similar dynamics are closer to each other. Some effects of heterochronic BMT are seen (left column), while the effects of aging are more clearly seen (more amplified colors in the right column) (Figure 2). Calculated statistically significant differences for the before mentioned parameters in Table 1, Table 2, Table 3, Table 4, Table 5, Table 6, Table 7 and Table 8 are also marked in this heatmap.

### 3.3. Frailty and Pathomorphological Changes

#### 3.3.1. Frailty

**Figure 3:**
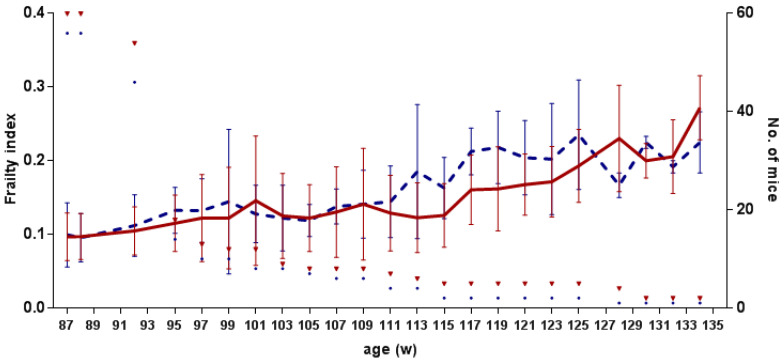
Frailty index (FI) results are shown in the combined plot. The *x*-axis shows the age of mice in weeks, the *y*-axis (**left**) shows FI (±SD) values, and (**right**) the number of analyzed mice. Legend: Solid line—BMT—transplanted aged group, dashed line—SHAM—aged control group, ▿—number of evaluated BMT mice, number of evaluated SHAM mice.

Four weeks after the last series of BM transplantation, we started the frailty evaluation with the noninvasive method of Whitehead et al. [23]. Every two weeks, from weeks 87 to 134, we evaluated 30 parameters and calculated the FI (Figure 3). In both groups, BMT and SHAM groups, FI was slowly increasing from an average of 0.097 ± 0.032 and 0.099 ± 0.044 to 0.272 ± 0.044 and 0.225 ± 0.042, respectively. FI between the transplanted and control group did not differ significantly (df = 102.82; F = 0.860; *p* = 0.356).

#### 3.3.2. Pathomorphological Changes

**Figure 4:**
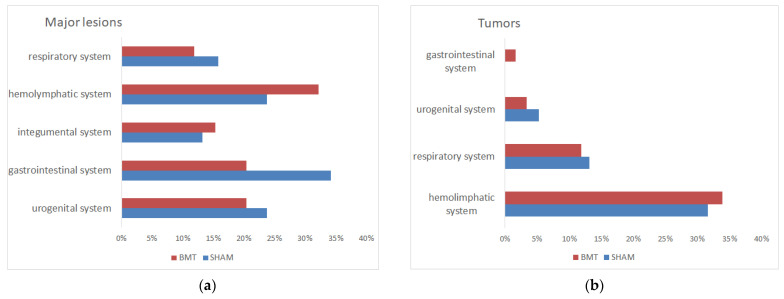
Results of pathomorphological analyses of the aged mice that received heterochronic BM transplantation (BMT, *n* = 59) and of the aged control mice (SHAM, *n* = 38); (**a**) the percentage of major lesions in different organ systems; (**b**) the percentage of tumors in different organ systems.

The predominant pathomorphological findings were tumors and inflammation in different organs and tissues (Figure 4a). The most often detected tumors were lymphomas followed by pulmonary adenocarcinoma or adenoma (Figure 4b). Differences in the major lesions or causes of death between the transplanted aged group and the aged control group were not significant, the heterochronic BM transplantation was not correlated to any of the groups.

### 3.4. Longevity

**Figure 5:**
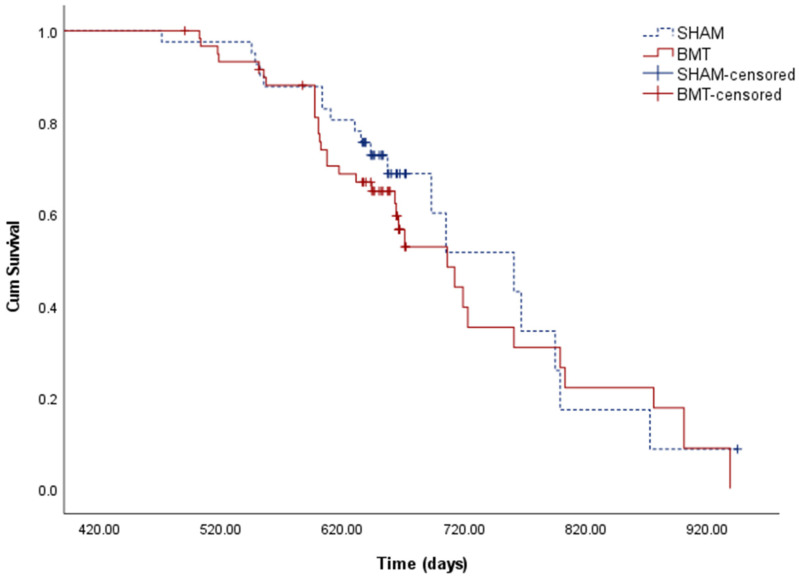
Kaplan–Meier survival curves. The equality test showed no significant differences between the transplanted group (BMT) and the control group (SHAM). BMT-censored and SHAM-censored are the mice that died during the transplantation process or were deliberately euthanized for the immune system analyses.

The longevity of mice was statistically evaluated by Kaplan-Meier survival analysis (log-rank test) (Figure 5). From 60 mice in the BMT group, 24 were censored, and from 41 mice in the SHAM group, 22 were censored. On average (median), mice lived 706 days, 95% CI (647, 764), in the BMT group and 761 days, 95% CI (662–859), in the SHAM group. The equality test showed no significant differences between the groups.

In the Cox proportional hazard regression analysis, the risk of death between the groups was not significantly different (B = 0.202 *p* = 0.480, HR = 1.224; CI = 0.699, 2.144).

## 4. Discussion

According to the stem cell and immune theories of aging, supplying an aged organism with a young hematopoietic stem cell repertoire should increase its regenerative and immune capacities with the resulting enhancement of general health, increase in immune functions, and a decrease in morbidity, and potential extension of life span.

In our study, we addressed the potential of such a heterochronic approach in a model of non-conditioned autologous HSCT in a murine setting, with the intent of observing its influence on the functions of the immune system, frailty, general health, and longevity. As already noted in the introduction, the actual autologous setting is practically not attainable in our model, so it was approximated by using a syngeneic heterochronic and heterosexual donor/recipient combination to closely mimic the autologous setting. In addition, pure HSCT was supplemented by transplantation of whole BM.

### 4.1. Chimerism after the Heterochronic BMT in Non-Conditioned Mice

With our protocol, female mice developed on average 18.7 ± 9.6% chimerism in the BM, or 0.15 ± 0.08% chimerism per one million transplanted BM cells, which is in accordance with previous studies on the BALB/c strain [18]. Chimerism was similar in neutrophils and B cells from the BM, whereas it was much lower in the spleen T cells, which could be due to age-associated thymic atrophy and a decrease in naïve T cell numbers [27,28]. Interestingly, there is more than a double level of chimerism in the proliferated cells in CFU assays compared to the chimerism in the native BM (46 ± 15% vs. 18.7 ± 9.6%). This phenomenon can be attributed either to a higher proliferative capacity of donor-derived committed progenitor cells in our in vitro setting, to a possible quiescence of donor HSPCs in the BM after the engraftment, but also to a possible natural suppressor activity of the recipient’s niche [29].

Besides the engraftment, we were interested in the possible expansion of the HSC number after the BMT. For this reason, we have also analyzed the HSC frequency in the BM, which are defined as Lin^−^c-Kit^+^, CD150^+^, CD48^−^, CD41^−^ cells, and the results did not show statistically significant HSC expansion in 21-month-old mice. Our results are from previous studies by [30,31].

In our model, there was no correlation between the number of transplanted BM cells and the number of HSCs per 100,000 total BM cells after engraftment in transplanted mice (data not shown). It seems that after the BMT, the donor’s young HSCs compete for space in the BM with the recipient’s HSCs, supporting the so-called replacement model of the engraftment [32].

### 4.2. Influence of the Heterochronic BMT on the Immune System

#### 4.2.1. Influence of the Heterochronic BMT on the Innate Immune System

In aged mammals, the most prominent immune defect is subclinical inflammation (so-called “inflamm-aging”) [33,34]. Another phenomenon is the increased number of myeloid cells, which is probably due to age-related myeloid-biased differentiation from the HSCs [35]. Heterochronic BMT could mitigate these negative phenomena. In our model, heterochronic BMT had a statistically significant effect on the relative neutrophil count in the spleen (2.68 ± 1.86% in the BMT and 1.86 ± 0.51% in the SHAM group, *p* = 0.015) and in the BM (27.4 ± 8.6% in the BMT group and 37.1 ± 3.7% for the SHAM group, *p* = 0.014). The relative neutrophil count in transplanted mice was similar to that in young mice (Y group relative neutrophil number in the spleen was 2.29 ± 0.89% and in the BM was 31.9 ± 12.6%). This result shows a possible rejuvenating effect of the heterochronic BM transplantation.

On the other hand, the endocytosis assay that was used to explore the expected improved capacity of inflammatory and non-inflammatory monocytes and neutrophils showed no statistically significant effect of the heterochronic BMT on endocytosis rates. Besides, we could not confirm any significant differences in the aged compared to young mice. This is unexpected since previous reports show that while the phagocytosis, chemotaxis, exocytosis, and ROS formation remain unchanged in the neutrophils of aged mice, the rates of phagocytosis should decrease [36,37]. We found no explanation for this fact.

In aged organisms, subclinical chronic inflammation is frequently present, and an increase in pro-inflammatory cytokines is seen in [38,39]. In our model, we performed a cytokine release assay, where we incubated cells from the spleen and peritoneal cavity with LPS and then measured IL-6, TNF, and MCP-1 in the supernatant, but found no statistically significant differences in the measured cytokines in the transplanted vs. control mice. However, there weren’t any significant differences between young and old mice due to aging effects. The latter was again unexpected, as in vivo stimulation of mice with LPS and other bacterial products should lead to a higher release of cytokines TNFα, IL-1β, and IL-6 in aged mice than in young mice [33,40].

#### 4.2.2. Influence of the Heterochronic BMT on the Adaptive Immune System

With age, B cell response to vaccinations and infections is decreased, B cells produce a lower number of antibodies, which are also of lower quality. B cells develop in the BM from HSCs and mature through different maturation stages: from pre-pro-B cells, through pro-B to pre-B, and immature B cells. The latter leaves the BM and mature in the spleen, where they can stay, enter the bloodstream, travel to other lymph nodes, or return to BM. Different strains of mice show various aging defects; most commonly, they are seen in the pre-B population, but also in the pro-B and immature B cell populations [41,42,43].

In our nonmyeloablative (non-irradiated) model of transplanting young HSCs to aged mice, we observed no impact on the recipient B cell maturation stages. In the conditioned BM transplantation, Hida et al. (2010) injected C57BL/6^CD45.1/CD45.1^ cells directly into the tibias of irradiated congenic C57BL/6^CD45.2/CD45.2^ recipients and observed a positive impact on pre-B, pro-B, immature, and mature B cell populations [44].

Interestingly, the relative counts of B cell populations were negatively correlated to the levels of achieved chimerism in the BM for the populations prepro-B (r = −0.623, *p* = 0.0305) and immature B cells (r = −0.607, *p* = 0.0365).

Similarly, there was no heterochronic BMT effect on the population of follicular B (FO B) cells, marginal zone B (MZ B) cells, and age-associated B cells (ABCs), isolated from the spleen. However, we documented a clear difference between the old (4.6 ± 2.9%) and young mice (1.2 ± 1.3%, *p* = 0.009) in the population of ABC cells. ABCs are a newly discovered population of mature B cells that accumulate gradually with age in the spleen and the BM and less frequently in the peritoneal cavity and the lymph nodes [45,46,47,48]. ABCs consistently differentiate from the FO B cells in the presence of proinflammatory molecules and pathogens. They probably affect different stages of B cell development through the negative feedback loop and lead to a lowered number of developed B cells [41,49] with the IL-21 and IFN-Υ released by the T cells and adipocytes being the most important cytokines for this process [41].

Besides conventional B cells (B2 cells), the peritoneal cavity also harbors B1 cells, which are first produced in the fetus, and later in life self-renew in the periphery. Their numbers also increase with age [41]. Our analysis of B2 and B1 subpopulations from the peritoneal cavity showed statistically significant effects of heterochronic BMT. There were 35.6 ± 15.6% and 56.0 ± 16.1% B2 cells (*p* = 0.016), and 64.4 ± 15.6% and 44.0 ± 16.1% B1 cells (*p* = 0.016) in the B cell population, in the BMT and SHAM group, respectively. In the B1 population, there were 62.7 ± 17.6% and 46.3 ± 5.1% B1a cells (*p* = 0.006), and 12.7 ± 2.9% and 22.4 ± 8.4% B1b cells (*p* < 0.001) in the BMT and SHAM group, respectively. The relative numbers of subpopulations B1a and B1b showed rejuvenating effects of the heterochronic BMT, while B1 and B2 populations showed an adverse effect of the heterochronic BMT. It appears that adverse effect is present in some B1 subpopulation that was not included in our analysis.

With age, levels of apoptosis increase, thymus involutes, T cell repertoire changes, the relative number of naïve T cells decreases, and the relative number of memory T cells increases [50]. Aging also significantly impacts T cell subpopulations such as Th cells, Tc cells, and Treg cells. Changes occur on the level of proliferative capacity and cytokine release, and the declines in T cell function arise from the decreased number of naïve T cells and accumulation of defective naïve and memory T cells, and intrinsic defects that occur in the Th and Tc cell signaling [51]. Correspondingly, aging changes the phenotype of T cells and their response kinetics [52]. In C57BL/6 mice, a decrease in Th numbers and an increase in Tc numbers with age were reported previously by [53].

In our study, we analyzed Th and Tc cells that are, by the presence of CD44 and CD62L markers, divided into subpopulations: naïve, central memory, effector memory, and acute/activated effector cells [54,55,56,57,58,59,60]. The effects of heterochronic BMT in aged mice were seen in the central memory Th cells (36.3 ± 10.5% BMT vs. 28.9 ± 13.8% SHAM, *p* = 0.040), in the effector/effector memory Th cells (63.3 ± 10.3% BMT vs. 71.4 ± 13.4% SHAM, *p* = 0.056) and in the effector/effector memory Tc cells (20.5 ± 10.1% BMT vs. 32.8 ± 30.7% SHAM, *p* = 0.033). These results show the rejuvenating effect of the heterochronic BMT in old mice.

Regulatory T cells, i.e., Tregs, are crucial for the immune system equilibrium. With their immune suppression ability, they influence many of the immune responses, prevent the development of autoimmune diseases, preserve pregnancy, and prevent exaggerated immune responses in the organ and tissue transplantations [61]. There is a relatively higher number of Treg cells in the aged mice than in young mice. They are also more capable of suppressing the immune system, which is, according to the inflamm-aging theory, due to the growing number of chronic inflammations [62,63]. An increase in Treg in the elderly may cause excessive suppression of immune responses and thus contribute to the immunosenescence [61]. In our study, we saw the aging effect in the Treg population (4.0 ± 1.4% old vs. 2.8 ± 1.6% young, *p* = 0.001). The heterochronic BMT did lead to a slightly lower relative number of Tregs, but the transplantation effect was not significant (3.4 ± 1.2%, *p* = 0.125).

In our study, the Pearson correlation analysis did not show any correlation between the relative number of different T cell populations and levels of chimerism achieved in recipient mice (data not shown).

A proliferation assay was performed in vitro, using PHA and PMA as stimulants to assess the effect of heterochronic BMT on lymphocyte proliferation. The lymphocytes of transplanted aged mice proliferated more than those of aged control mice but did not reach full statistical significance (*p* = 0.056). It is known that the lymphocyte proliferation ability in mice and humans decreases with age [50,64,65]. While some authors have found substances that influence lymphocyte proliferation in aged people, e.g., vitamin C, vitamin E, and NADH, our study is the first to show the (borderline) positive influence of heterochronic BMT in doing so in non-conditioned aged mice. Whether this is due to the higher proliferation of all lymphocytes or only donor-derived lymphocytes is unclear and should be confirmed after the assay with chimerism analysis.

To test the impact of heterochronic BMT on the functional capacity of the recipient’s immune system, we performed an in vivo immunization with the KLH antigen, a T cell-dependent antigen. Quantification of immunization response is an indirect method that reflects multiple aspects of immunocompetence, such as processing and presentation of antigen, surface receptor expression, cytokine release, somatic hypermutation, and immunoglobulin class switching. The activity of many of these mechanisms decreases with age (e.g., T cells release less IL-2, proliferate and differentiate less, express less CD40L, …), leading to a lesser quantity and quality of the developed antibodies. A similar age-related lower amount of antibodies against KLH has already been shown in mice [66] and humans [67].

In our study, the aged mice showed the effects of aging and had a lower relative amount of IgG antibodies in the serum than the young ones (*p* = 0.0015); however, there was no difference between the transplanted and control mice. We have to note that we used FCA and FIA in our immunization study, which is known for their uncontrolled immune-boosting and altering of auto- and alloimmune responses, and these could possibly hide the transplantation effect.

### 4.3. Influence of the Heterochronic BMT on the Frailty and Pathomorphological Changes

Frailty is, by definition, a state of increased vulnerability to adverse health outcomes for older adults of the same chronological age [68]. We evaluated the frailty with the frailty index (FI), which calculates health deficiencies of numerous organ systems in the body [69]. This laborious method uses 30 clinical signs to evaluate the integument, physical/musculoskeletal system, vestibulocochlear/auditory system, digestive/urogenital system, respiratory system, discomfort, temperature, and weight [23]. According to our hypothesis, the group of transplanted aged mice should present a lower FI than the control group; however, the results showed no statistically significant differences.

Pathomorphological analysis showed that the mice in our study developed different types of tumors (40–47%), diseases of the urogenital system (17%, including vaginal and uterine prolapse, cystolithiasis/bladder chronic inflammation, endometritis, kidney amyloidosis, and endometrial hyperplasia), diseases of the gastrointestinal system (16%, including gastroenteritis, hepatitis, liver abscess, and rectal prolapse), and the diseases of the integumental system (14%—chronic granulomatous panniculitis/myositis). Some mice had no visible lesions (3–5%). Tumors were mostly detected in the haemolymphatic system (63–67%—lymphoma and granulocyte leukemia), the respiratory system (23–26%—lung adenoma/adenocarcinoma), the urogenital system (7–11%—ovary adenocarcinoma and mammary adenocarcinoma) and the gastrointestinal system (0–3%—salivary gland adenocarcinoma). There were no significant differences among detected lesions between the transplanted and control groups.

Common diseases listed by other authors for BALB/c mice are similarly the mammary gland tumors (30% at the age of two years) [70], kidney tumors (25–48%), reticuloendothelial tumors (11–20%), lung tumors (10–16%), synoviomas (2–8%) [71] and heart failure (17–62%) [72].

### 4.4. Influence of the Heterochronic BMT on the Longevity

Various studies expanded the lifespan of the mice with rapamycin, resveratrol, metformin, sirtuin activators, and caloric restriction approach [73,74], and only a few used the transplantation of young stem and progenitor cells. Various sources and types of these cells were used, such as in vitro grown mesenchymal stem cells (MSCs) from the murine BM [75,76] or the human adipose tissue [77,78], muscle stem and progenitor cells [79], and freshly isolated BM cells or HSCs [80,81,82,83]. The lifespan was reportedly extended in various experiments [75,80,81,83,84,85]. In our model, the repeated heterochronic heterosexual syngeneic BMT did not extend the lifespan of transplanted mice, which lived 706 days (647–764 days) compared to the 761 days (662–859) of the control mice (median, 95% CI). Moreover, the transplanted BM cells did not have a statistically significant effect on the risk of death.

Although the heterochronic non-myeloablative BMT procedure significantly improved individual parameters of the immune system, the total sum of antiaging effects wasn’t enough to extend the longevity in our model setting. We assume several possible explanations for this phenomenon, primarily due to the complexity of such in vivo animal study.

The idea of heterochronic HSC autotransplantation after long-term cryopreservation of HSCs is impossible to test in humans in a clinical trial. It is even hard to test it in the mouse model due to the technical and biological requirements. In our model, we used young male BALB/cAnNCrl mice as donors and females of the same strain as recipients, as there is a negligible immunoreactivity to the minor Y chromosome-associated histocompatibility antigens [15,16,17], resulting in a setting where the donors and recipients still did not match completely; therefore the minor histocompatibility antigens (miHAs) associated with the Y-chromosome could have caused subtle immune responses [86].

On average, 18.7% donor chimerism was achieved in our non-myeloablative setting, which in theory should have been high enough for rejuvenating effects [18]. Higher chimerism would be possible with prior conditioning, which is not consistent with the basic idea of haHSCT.

One major player in our setting might have critically influenced the modest rejuvenation success of our model, namely the HSC niche. The HSC niche is a complex and dynamic interaction of many cell types, such as endothelial cells, MSCs, osteoblasts, adipocytes, neuroglia, and mature hematopoietic cells. The HSC niche ages along with the organism, the niche cells change, senescence of the microenvironment occurs, MSC differentiation becomes unbalanced, there appears vascular remodeling, changes in adrenergic signaling, increase in inflammation, and disruptions of endothelium-instructive function [87], resulting in microenvironmental remodeling, that prevents the optimal regulation of HSCs and their progeny [88,89,90]. In other words, an old HSC niche could actively prevent the successful transplantation of young HSCs. Interestingly, when HSCs of old mice were transplanted to young mice, they discovered that the gene expression profile of the aged HSCs became similar to the profile of the young HSCs; however, the functional defects of HSCs did not renew [91].

Another major player that could impede the overall success of haHSCT in our experiment is the aging of the thymus, an immunologically weak point of any cellular-based anti-aging intervention. The aging thymus does not provide sufficient numbers of fresh naive T-cells, and it is also known to resist non-specific rejuvenating attempts. On the other hand, there is ample evidence that transplanted cells after autologous HSCT migrate to and populate the thymus, which is even enhanced after serial transplantations [17,92,93,94,95,96,97]. Stem cells from various sources seem to finally repair the damaged or old thymus, and this colonization maintains intact thymocytic niches and regenerates various thymic functions [98,99,100,101,102]. Although the young thymus was not co-transplanted in our setting, we presumed that haHSCT could act beneficially on thymic function, comprising the regeneration of naïve B cells and a stable thymic reactivation with re-emergence of thymic-derived naïve T cells, which was only partly achieved.

## 5. Conclusions

In conclusion, our model of the heterochronic non-myeloablative transplantation resulted in specific rejuvenation effects on some immune cell subpopulations, i.e., neutrophils in the spleen and BM, central memory Th cells, effector/effector memory Th cells, and effector/effector memory Tc cells in the spleen, and B1a and B1b cells in the peritoneal cavity. It also enhanced the proliferation capacity of lymphocytes with borderline significance. However, these rejuvenating effects were too restricted to be conveyed into expanding the life span, which can be due to several limitations. There are some possible drawbacks in the design of our experimental animal model, which open several further scientific questions. The first one is about a lack of complete donor/recipient tissue histocompatibility arising from heterosexual transplantation and the fact that the recipients received cells from eight different donors, which could cause some subclinical chronic long-term transplantation related chronic rejection due to the Y-chromosome based minor histocompatibility antigens or other antigens, although negative mixed lymphocyte reactions initially excluded such effects, and no signs of rejection or graft versus host disease were seen in the post-mortem analyses. Another critical question is whether the relatively limited degree of donor chimerism obtained (18.7% on average) ranges below the critical proportion of young stem cells that could initiate a significant immune and hematological rejuvenation of the aged animals. The next possible question is about the nature of the old HSC niche. It seems that the old niches develop a particular state of impeding the development of transplanted HSC progeny, thereby demanding specific conditioning steps for successful engraftment. Finally, there remains an unanswered question of epigenetic differences in the current setting, where the epigenetic state of young BM cells could interfere with the epigenetic state of an aged organism, which could lead to not yet explained immune disharmony. We hope that these questions will soon be addressed and answered in the following stages of similar research.

## Figures and Tables

**Table 1:** Immunophenotyping by flow cytometry (cell source: spleen).

Cell Populations in the Spleen *n* (BMT) = 11, *n* (SHAM) = 12	Heterochronic BMT Effects
CD11b+Ly6G-monocytes/macrophages	ns
CD11b+Ly6G-Ly6Chi inflammatory monocytes	ns
CD11b+Ly6G-Ly6C-/lo non-inflammatory monocytes	ns
CD11b+Ly6G+ neutrophils	⊕ *
CD19+CD21/35-CD23-CD43-AA4.1-ABCs	ns
CD19+CD21/35+CD23+CD43-AA4.1-FO B cells	ns
CD19+CD21/35+CD23-CD43-AA4.1-MZ B cells	ns
CD3+ T cells	ns
CD3+CD4+ Th cells	ns
CD3+CD4+CD44-CD62L+ naïve Th cells	ns
CD3+CD4+CD44+CD62L+ central memory Th cells	⊕ *
CD3+CD4+CD44+CD62L- effector/effector memory Th cells	⊕ ^(^*^)^
CD3+CD8a+ Tc cells	ns
CD3+CD8a+CD44-CD62L+ naïve Tc cells	ns
CD3+CD8a+CD44+CD62L+ central memory Tc cells	ns
CD3+CD8a+CD44+CD62L- effector/effector memory Tc cells	⊕ *
CD3+CD8a+CD44-CD62L-acute/activated effector cells Tc cells	ns
CD4+FoxP3+	ns
CD4+FoxP3+CD25+ Treg	ns

Legend: ⊕ rejuvenating effect, ^(^*^)^—*p* = 0.056, *—*p* < 0.05, ns—not significant.

**Table 2:** Immunophenotyping by flow cytometry (cell source: bone marrow).

Cell Populations in the Bone Marrow *n* (BMT) = 12, *n* (SHAM) = 12	Heterochronic BMT Effects
CD11b+Ly6G+ neutrophils	⊕ *
CD11b+Ly6G- monocytes/macrophages	ns
B220loCD43+CD19-sIgM-sIgD-prepro-B	ns
B220loCD43-CD19+sIgM-sIgD-pre-B	ns
B220loCD43+CD19+sIgM-sIgD-pro-B	ns
B220loCD43-CD19+sIgM+sIgD-immature B cells	ns
B220hiCD43-CD19+sIgM+sIgD+ mature B cells	ns
B220+ B cells	ns

Legend: ⊕ rejuvenating effect, *—*p* < 0.05, ns—not significant.

**Table 3:** Immunophenotyping by flow cytometry (cell source: peritoneal cavity).

Cell Populations in the Peritoneal Cavity *n* (BMT) = 8, *n* (SHAM) = 6	Heterochronic BMT Effects
B220+ B cells	ns
B220-CD23+ B2 cells	⊝ *
B220+CD23- B1 cells	⊝ *
B220+CD23-CD11b+CD5+ B1a cells	⊕ *
B220+CD23-CD11b+CD5- B1b cells B220-CD11b+ monocytes/macrophages	⊕ ** ns

Legend: ⊕ rejuvenating effect, ⊝ adverse effect, *—*p* < 0.05, **—*p* <0.01, ns—not significant.

**Table 4:** In vitro endocytosis evaluation (cell source: spleen).

In Vitro Endocytosis Evaluationn *n* (BMT) = 12, *n* (SHAM) = 13	Heterochronic BMT Effects
CD11b+Ly6G-Ly6Chi inflammatory monocytes	ns
CD11b+Ly6G-Ly6C-/lo non-inflammatory monocytes	ns
CD11b+Ly6G+ neutrophils	ns

Legend: ns—not significant.

**Table 5:** In vitro cytokine release assay (cell source: spleen).

In Vitro Cytokine Release Assay, Spleen Cells *n* (BMT) = 13, *n* (SHAM) = 13	Heterochronic BMT Effects
IL-6	ns
TNF	ns

Legend: ns—not significant.

**Table 6:** In vitro cytokine release assay (cell source: peritoneal cavity).4285.

In Vitro Cytokine Release Assay, Peritoneal Cells *n* (BMT) = 10, *n* (SHAM) = 10	Heterochronic BMT Effects
IL-6	ns
MCP-1	ns
TNF	ns

Legend: ns—not significant.

**Table 7:** In vitro splenocyte proliferative response (cell source: spleen).

In Vitro Splenocyte Proliferative Response *n* (BMT) = 15, *n* (SHAM) = 15	Heterochronic BMT Effects
PHA	ns
PHA+PMA	⊕ ^(^*^)^

Legend: ⊕ rejuvenating effect, ^(^*^)^—*p* = 0.056, ns—not significant.

**Table 8:** In vivo functional testing.

In Vivo Immunization *n* (BMT) = 9, *n* (SHAM) = 10	Heterochronic BMT Effects
Absorbance at 10^5^ titre	ns

Legend: ns—not significant.

## Supplementary Materials

The following supporting information can be downloaded at: https://www.mdpi.com/article/10.3390/biom12040595/s1. Figure S1: Flow cytometry gating strategy for neutrophils, monocytes/macrophages, inflammatory and non-inflammatory monocytes, Figure S2: Flow cytometry gating strategy for ABCs, FO B cells and MZ B cells, Figure S3: Flow cytometry gating strategy for Th and Tc cells, Figure S4: Flow cytometry gating strategy for Treg cells, Figure S5: Flow cytometry gating strategy for prepro-B, pre-B, pro-B, immature and mature B cells, Figure S6: Flow cytometry gating strategy for B-2 and B-1 cells; Table S1: Source data for the heatmap.
