# Supplementary material for: The Influence of Heterochronic Non-Myeloablative Bone Marrow Transplantation on the Immune System, Frailty, General Health, and Longevity of Aged Murine Recipients
Source: Biomolecules. 2022 Apr 18;12(4):595. doi: 10.3390/biom12040595 (PMC9028083; doi:10.3390/biom12040595)
Supplement: Supplementary file 1 [file biomolecules-12-00595-s001.zip › Supplementary Figures S1-S6.pdf]

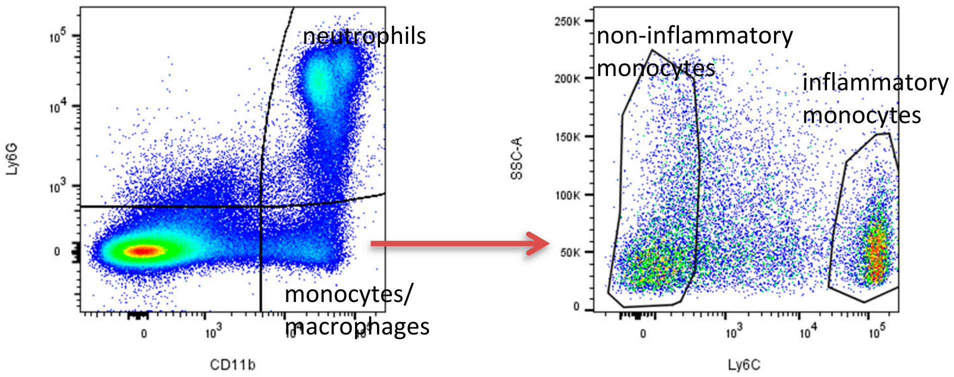

Figure S1: Flow cytometry gating strategy for neutrophils, monocytes/macrophages, inflammatory and non-inflammatory monocytes. Analyzed cells were live single cells (FSC/SSC, SSC-H/SSC-W, FSC-H/FSC-W, and 7-AAD/SSC-A dot plots are not shown).

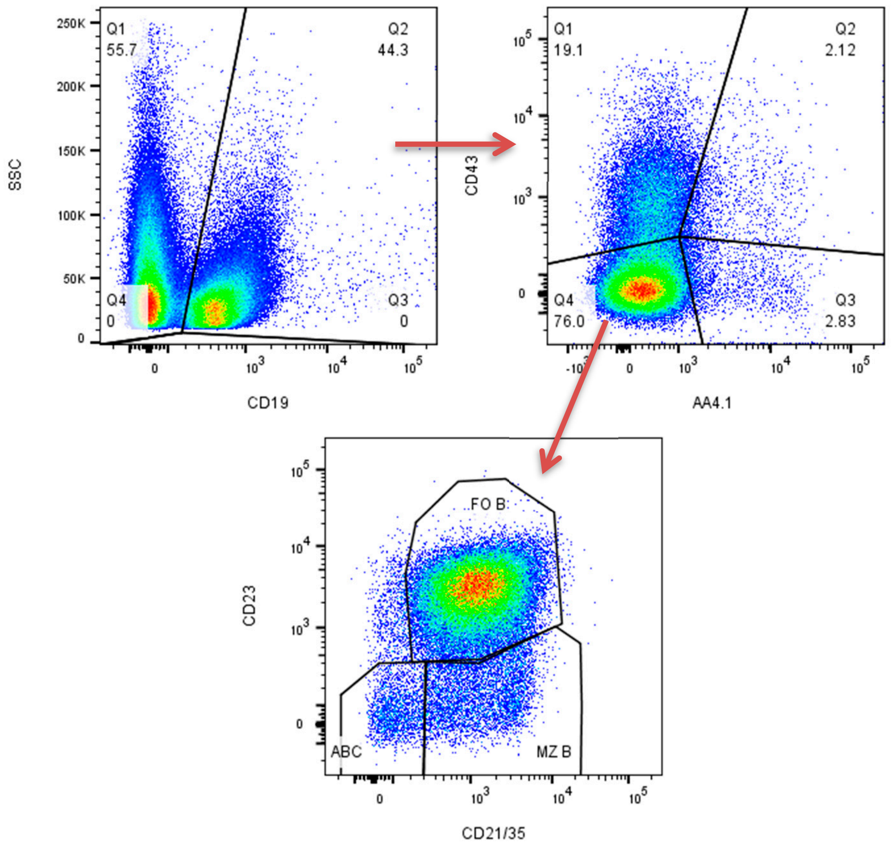

Figure S2: Flow cytometry gating strategy for ABCs, FO B cells and MZ B cells. Analyzed cells were live single cells.

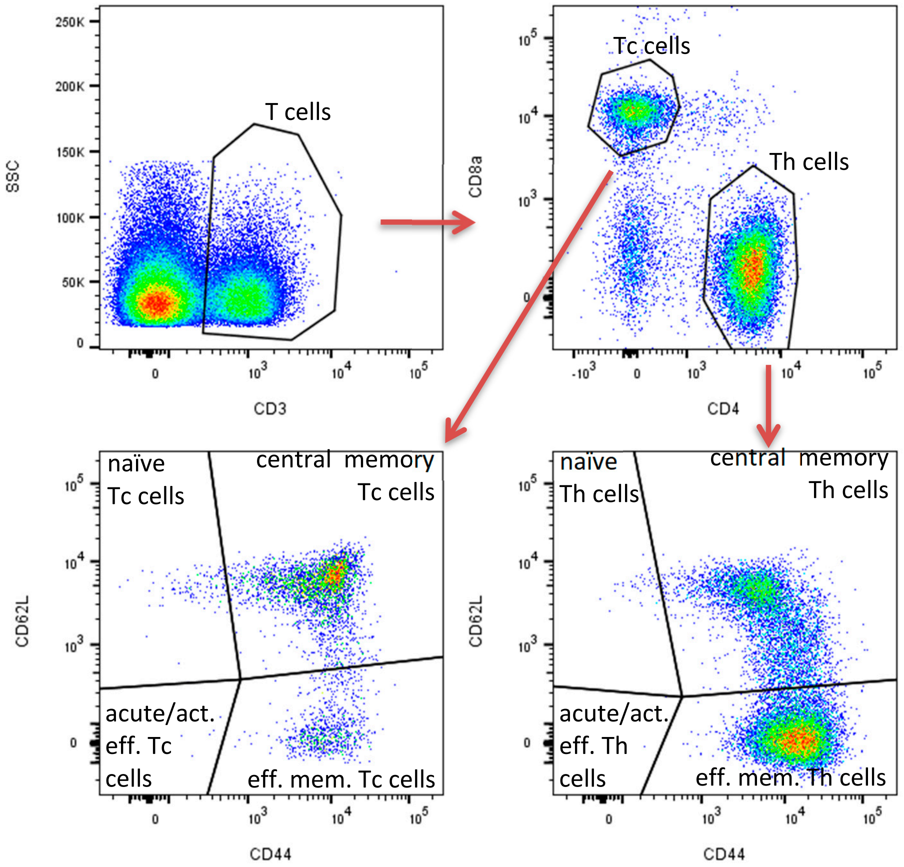

Figure S3: Flow cytometry gating strategy for Th and Tc cells. Analyzed cells were live single cells.

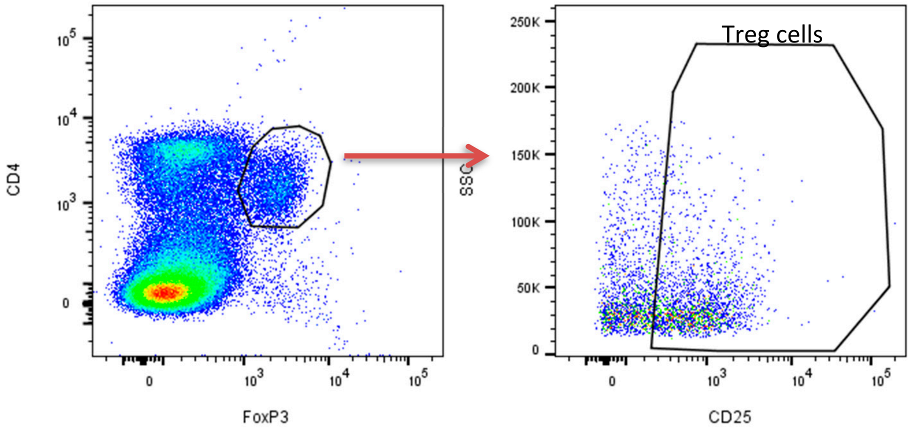

Figure S4: Flow cytometry gating strategy for Treg cells. Analyzed cells were single cells.

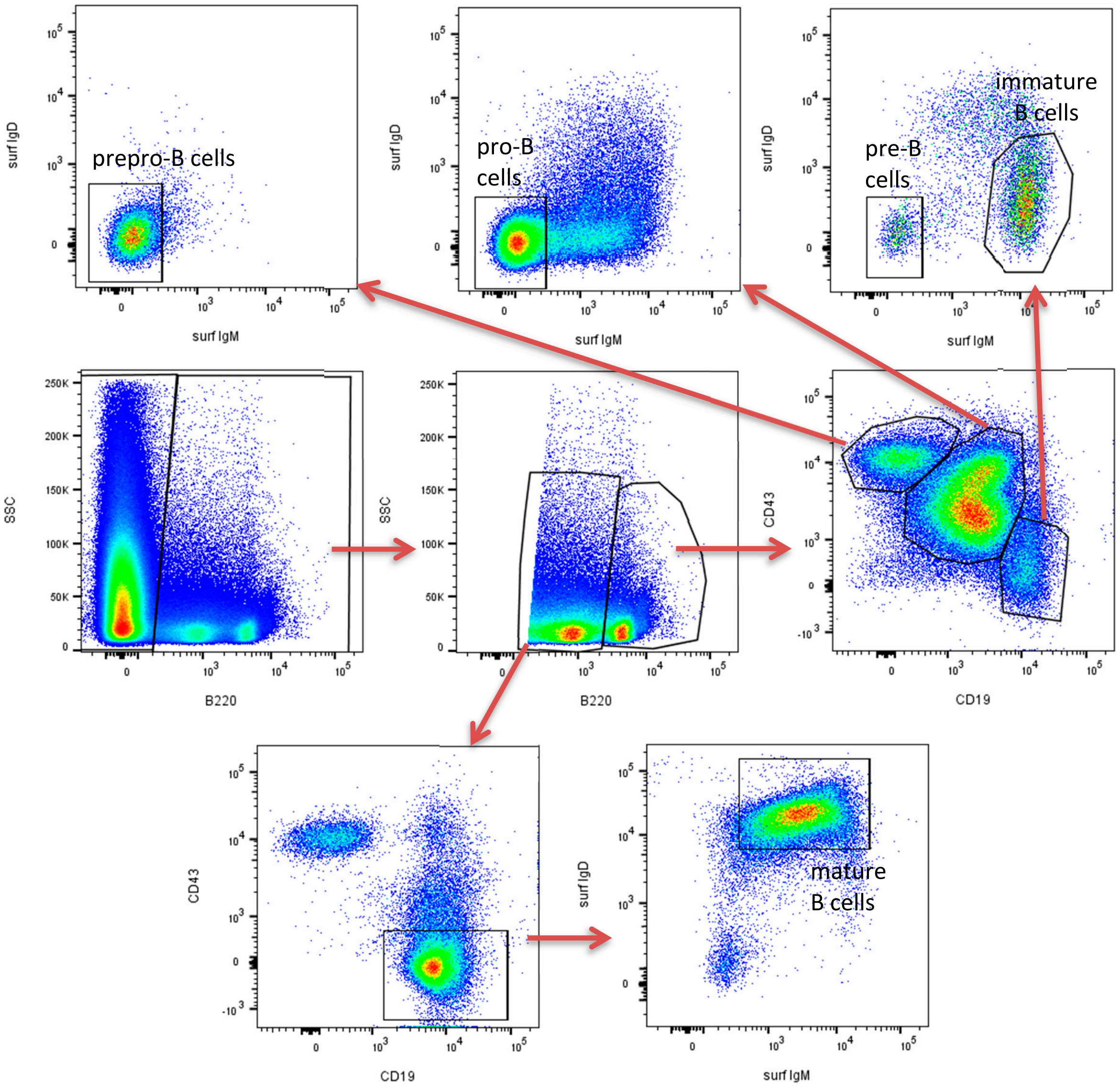

Figure S5: Flow cytometry gating strategy for prepro-B, pre-B, pro-B, immature and mature B cells. Analyzed cells were live single cells.

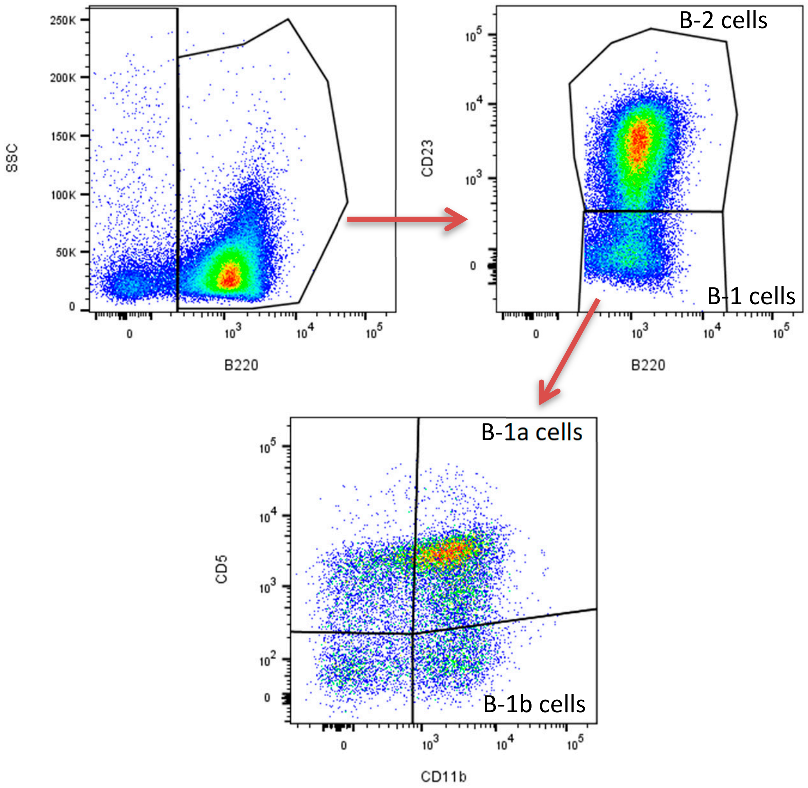

Figure S6: Flow cytometry gating strategy for B-2 and B-1 cells. Analyzed cells were live single cells.
