# Supplementary material for: The Influence of Heterochronic Non-Myeloablative Bone Marrow Transplantation on the Immune System, Frailty, General Health, and Longevity of Aged Murine Recipients
Source: Biomolecules. 2022 Apr 18;12(4):595. doi: 10.3390/biom12040595 (PMC9028083; doi:10.3390/biom12040595)
Supplement: Supplementary file 1 [file biomolecules-12-00595-s001.zip › Table S1.pdf]

Table S1. Source data for the heatmap.

FinalName	BMT	SHAM	Y	%CV	BMT vs SHAM	Y vs SHAM
spleen, monocytes/macrophages [%]	4,86	3,83	6,98	24%	0,34	0,87
spleen, monocytes, inflammatory [%]	15,08	16,62	6,73	45%	-0,14	-1,30
spleen, monocytes, non-inflammatory [%]	56,74	53,81	54,83	8%	0,08	0,03
spleen, neutrophils [%]	2,68	1,86	2,29	77%	0,53	0,30
spleen, monocytes, non-inflammatory [MFI]	1,901915	1,7982858	2,0559477	18%	0,08	0,19
spleen, IL-6 [cCK, pg/ml]	22,72578	32,6103	12,691613	61%	-0,52	-1,36
spleen, TNF [cCK, pg/ml]	11,06717	14,65864	16,8908	73%	-0,41	0,20
BM, neutrophils [%]	27,4	37,1	31,9	28%	-0,44	-0,22
BM, monocytes/macrophages [%]	8,9	11,2	10,7	11%	-0,33	-0,07
peritoneal cavity, monocytes/macrophages [%]	19,5	24	30,7	47%	-0,30	0,36
proliferation, control [cGy]	1474,987	1597,133	437,307	75%	-0,11	-1,87
proliferation, PHA [cGy]	3367,627	4103,253	5211,063	51%	-0,29	0,34
proliferation, PHA+PMA [cGy]	26524,7	21003,14	43449,12	30%	0,34	1,05
HSC / live cell no.	0,0000864	0,0000757	0,0000578	19%	0,19	-0,39
BM, B cells [%]	23,3	25,2	17,4	25%	-0,11	-0,53
B cells, mature [%]	14,4	11,8	3,9	54%	0,29	-1,60
B cells, prepro-B [%]	4,9	3,6	7,6	24%	0,44	1,08
B cells, immature [%]	1,5	1,1	0,2	63%	0,45	-2,46
B cells, pre-B [%]	0,3	0,3	0,4	39%	0,00	0,42
B cells, pro-B [%]	42,3	46,1	60	18%	-0,12	0,38
spleen, B cells [%]	58,3	61,2	51,5	8%	-0,07	-0,25
B cells, ABC [%]	5,8	4,6	1,2	59%	0,33	-1,94
B cells, FO B [%]	85,4	84,5	89,1	3%	0,02	0,08
B cells, MZ B [%]	7,1	9	9,2	25%	-0,34	0,03
peritoneal cavity, B cells [%]	73,9	80,3	72	33%	-0,12	-0,16
B cells, B1 [%]	64,4	44	39,6	23%	0,55	-0,15
B cells, B-1a [%]	62,7	46,3	56	19%	0,44	0,27
B cells, B-1b [%]	12,7	22,4	18	29%	-0,82	-0,32
B cells, B-2 [%]	35,6	56	60,4	32%	-0,65	0,11
T cells [%]	30	30,5	34,1	7%	-0,02	0,16
Th cells [%]	62,2	65,1	64,4	10%	-0,07	-0,02
Th cells, acute/effector [%]	0,2	0,4	0,1	127%	-1,00	-2,00
Th cells, central memory [%]	36,3	28	66,1	40%	0,37	1,24
Th cells, effector [%]	63,3	71,4	33,3	29%	-0,17	-1,10
Th cells, naive [%]	0,2	0,2	0,6	51%	0,00	1,58
Tc cells [%]	21	20,4	24,6	14%	0,04	0,27
Tc cells, acute/effector [%]	0,9	0,7	0,7	36%	0,36	0,00
Tc cells, central memory [%]	75,8	65,1	77,2	8%	0,22	0,25
Tc cells, effector [%]	20,5	32,8	15,8	27%	-0,68	-1,05
Tc cells, naive [%]	2,8	1,3	6,2	61%	1,11	2,25
CD4+FoxP3+ [%]	3,4	4	2,8	23%	-0,23	-0,51
CD4+FoxP3+CD25+ [%]	63,3	66,4	65,4	14%	-0,07	-0,02
